# The Rehabilitation Interventions and Adaptive Technologies Used for Treating Patients With Cerebral Palsy

**DOI:** 10.7759/cureus.49153

**Published:** 2023-11-20

**Authors:** Alisha Guru, Aryan S Yadav, Tushar Sontakke

**Affiliations:** 1 Medical Education, Jawaharlal Nehru Medical College, Datta Meghe Institute of Higher Education and Research, Wardha, IND; 2 Medicine, Jawaharlal Nehru Medical College, Datta Meghe Institute of Higher Education and Research, Wardha, IND

**Keywords:** activity, kinesiology, physicians, ankle foot orthoses, botulinum toxin, physiotherapies, muscle strength, adaptive interventions, cerebral palsy

## Abstract

Cerebral palsy (CP) is one of the most common disorders in pediatric patients. The prevalence of CP is 2-3 in 1,000 live births, but various changes in some trends are seen in different groups. This article is a systematic review of multiple sources available for interventions and new adaptive techniques used for treating patients for their better lifestyles. With recent advancements, it is possible to diagnose a child who is below six months to two years. For achieving goals, proper interventions and techniques are necessary in the early stages of the disease. This article summarizes the rehabilitation and interventions available for treating these children with the best procedures.

## Introduction and background

Cerebral palsy (CP) is a heterogeneous group of disorders of movements and postures that have caused damage to the developing brain of the child. Apart from these, the patient also has difficulty communicating with other people, behavioral changes, hearing difficulties, vision changes, and life-threatening comorbidities. In addition, this patient also complains of pain at various sites and difficulty sleeping. The prevalence of CP in developed countries is 1.4-1.8 in 1,000 live births, with the majority being 2.95-3.2 per 1,000 live births [1]. It was earlier believed that lack of oxygen is mainly responsible for the still-developing brain of children with CP. With recent advancements, other causes and risk factors have also been identified; it is now thought that CP results from a series of events that cause injury to the developing brain of these children. Damage to the developing brain occurs before, during, or after birth and affects the neurological and musculoskeletal systems of patients with CP. Sensory disturbances with behavioral issues in the patient accompany abnormal muscle contractions, change in posture, and limitations in activity. Different strategies and prevention methods have been identified to decrease the occurrence of this disorder [2]. Therapeutic interventions for children with CP have drastically improved over the past 10 years. Different frameworks such as the World Health Organization's International Classification of Functioning, Disability and Health (ICF) have changed the scenario of treating patients with CP. These organizations primarily focus on providing a better lifestyle to the individual rather than treating their underlying symptoms and disabilities with an aspiration of improving their functions. Interventions for these children primarily focus on training exercises, crucial real-world responsibilities, and specifically aiming for their full engagement within the community [3].

The review is undertaken to assess the interventions and therapies for treating these diseases commonly affecting children. Their risk factors and pathologies have been reviewed, which may help in the interventions for treating CP patients.

## Review

Methodology

**Figure 1:**
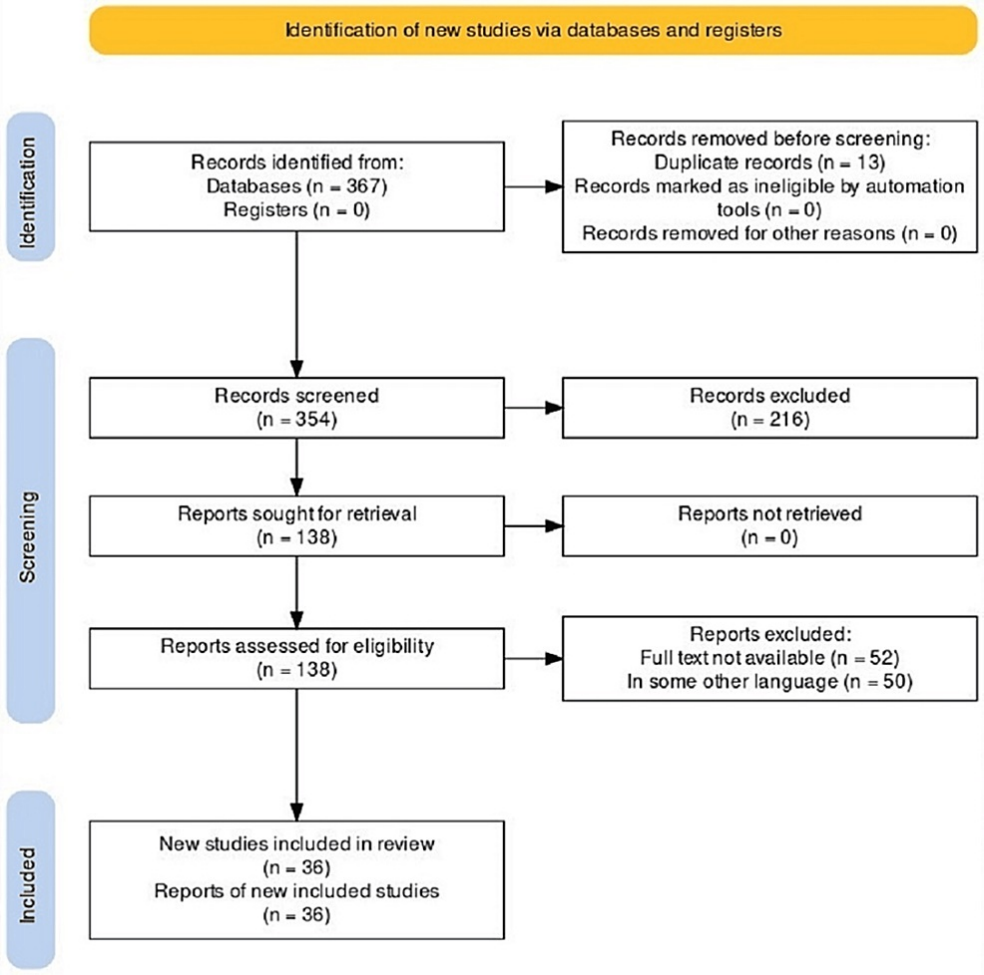
PRISMA flow diagram of the identification process for the sample of 36 articles included in this review PRISMA: Preferred Reporting Items for Systematic Reviews and Meta-Analyses

The strategies used for this review include considering research articles published in journals indexed in reputed, reliable, and authentic sources available on various platforms, processing reports according to different systems, and framing the review like a discussion section of an article where details are explained in straightforward sentences. The databases searched were PubMed, Google Scholar, and Web of Science. Review articles published within 10-15 years were included for review. This review adhered to the Preferred Reporting Items for Systematic Reviews and Meta-Analyses (PRISMA) guidelines. Inclusion criteria included CP causes, treatment, and interventions, while exclusion criteria included malignancy, comorbidities, and cardiovascular syndromes. The key terms used for the search are ("cerebral palsy "[Title/Abstract] OR "cerebral palsy "[Title/Abstract] OR "spasticity "[Title/Abstract] OR "techniques"[Title/Abstract]) AND ("botulinum toxin "[Title/Abstract] OR "Botox"[Title/Abstract] OR "BoNT-A"[Title/Abstract]) AND ("orthotic "[Title/Abstract] OR "orthoses"[Title/Abstract] OR "ankle foot"[Title/Abstract]) AND ("interventions "[Title/Abstract] OR (("decrease"[All Fields] OR "decreased"[All Fields] OR "decreases"[All Fields] OR "decreasing"[All Fields]) AND "electromyography"[Title/Abstract]) OR "decreased activity of muscles "[Title/Abstract] OR "robotics"[Title/Abstract]). Figure 1 summarizes the screening process and the number of articles in the final review.

CP disorder occurs in two to three out of a thousand live births. This disease is due to multiple causes of injury to the brain that affect postural balance and various body movements. The disorders associated with activities in patients with CP are dyskinesia (involuntary movements of the face, arms, legs, or trunk), ataxia (impaired coordination), or mixed type. Spasticity is the most common disorder associated with CP. It occurs in 70%-80% of cases of CP. It can also result in pain in the hip region or dislocation of the hip joint, balancing difficulties in patients, and various dysfunctions [4]. Mortality risks are associated with increased impairment due to the disorders. Table 1 shows the prevalence of CP in developing and developed countries.

**Table 1:** Prevalence of CP in developing and developed countries CP: cerebral palsy

	Developing countries	Developed countries
Prevalence of CP in 1,000 live births	2-5/1,000 live births	1.5-2/1,000 live births

Clinically, the diagnosis of CP is typically based on the observations of the doctors and the history of achievement of milestones of children given by parents, such as neck holding/neck control, trunk control (rollover), sitting with support, sitting without support, crawling, walking, reflexes of deep tendons, and tone of muscles [5]. Recent advancement shows various changes in the treating modalities in patients with CP, providing them with newer techniques and more effective interventions for better results. Effective prevention strategies for treating these patients include corticosteroids given in the antenatal period for the maturity of the lungs, magnesium sulfate (MgSo4) assigned to the mother, and prevention of hypothermia and hypoglycemia. Interventions include various physiotherapies, constraint-induced movement therapy, hippotherapy, botulinum toxin therapy, casting, correcting scoliosis using braces, umbilical cord therapy, and stem cell therapy [6].

**Figure 2:**
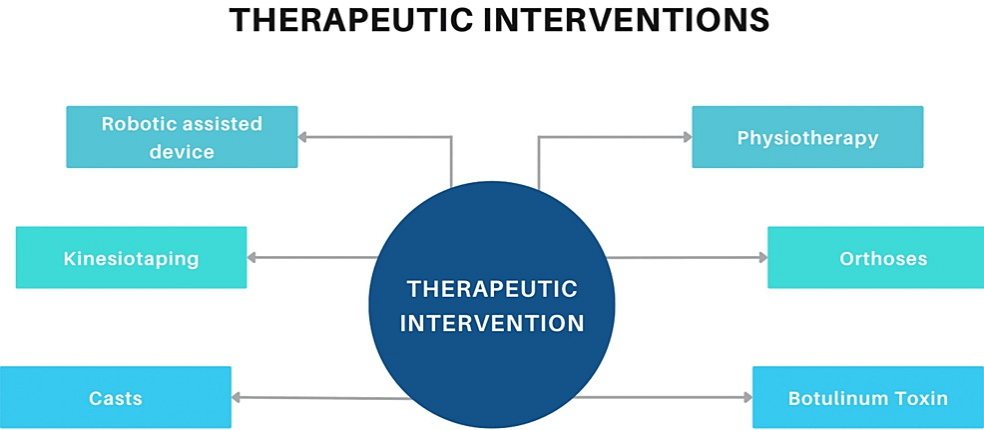
Therapeutic interventions for CP CP: cerebral palsy

Corticosteroids help prevent immaturity of the lungs in patients with CP. Before 36 weeks of gestation, corticosteroids are administered during the prenatal period. Nearly all individuals diagnosed with CP receive physiotherapies for many years. Treatment cannot impact the patient if it does not produce significant changes in the activity of the patients, improving their lifestyles, making them independent, and improving the quality of their health. The strategies that have been identified to have beneficial effects on impairments at the level of the body's structural capacity for activity are increasing the strength of the muscles by training the muscles, increasing the performance of the muscles by intense training, therapeutic hippotherapy on the symmetry of muscles, and training the patient to counterbalance. The desired improvement will require a longer period to impact the muscles significantly. This requires immense training and support from the doctors, family, and trainers [7]. Figure 2 shows the therapeutic interventions for CP.

Role of physiotherapy in treating patients with cerebral palsy (CP)

**Figure 3:**
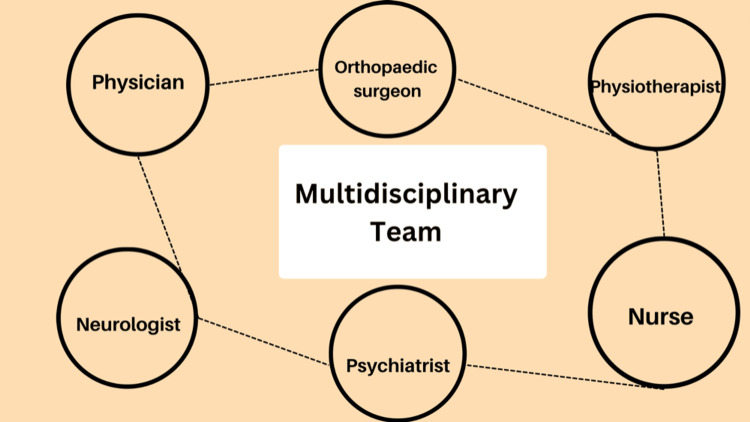
Multidisciplinary team for treating patients with CP CP: cerebral palsy

Children with CP spend their childhood receiving physiotherapies for training and strengthening the muscles to control gait performance and improve their quality of living [8]. All forms of resistance exercise, including plyometrics, power training, and strength training, are referred to as resistance training [9,10]. Long-term gross motor development has been demonstrated to be hindered by muscle contractures, but gross motor development is enhanced by intense exercise (around three times per week). Children are advised to do the following: five minutes of walking on a treadmill, riding a bicycle, physical stretching of muscles, bilateral stretching of hamstring muscles, doing squats, and stepping up stairs. To maintain strength, stability, and dynamic control in both the mid-stance and terminal swing face, adequate active terminal knee extension is crucial [8]. Nowadays, the use of external gym equipment such as weight machines, dumbbells for increasing muscle strength, treadmills for warmups, and electrical stimulation devices is rising tremendously. Although stretching and physiotherapy play a significant role in achieving the desired patient impact, it is more effective with interventions such as dynamic or static ortho strategies, botulinum toxin injections, or other spasticity-reducing medications or surgeries with manual stretching techniques. A team of doctors, such as psychiatrists, neurologists, developmental pediatricians, and orthopedists, typically manage motor impairments in children with CP (Figure 3) [9]. Some research shows that adding neurological music therapies also improves motor activity in children with CP. Neuromuscular electrical stimulation (NMES) is a common type of stimulation used along with other conventional treatments to improve motor function activities in children [11]. NMES also effectively controls the postures of children with CP. It supports the spine and back muscles and helps in sitting. It also has other advantages: it is a noninvasive technique, improves muscles, and helps proprioception [12].

Kinesiology taping technique

The kinesiology taping technique is gaining popularity in CP patients, and it is given as an adjuvant therapy with other interventions for the desired outcome. It is offered mostly for controlling the back posture of the child and improving sitting control. It is speculated that it enhances the functions of the muscles and myofascial fibers of the skeletal muscles. The constant afferent stimulation impacts mechanoreceptors, which allows more sensory signals to flow in the nervous system for the proper functioning of the muscles and thus provides voluntary control and coordination in muscles [13]. Kinesiology taping offers remarkable improvements in managing pain, and it is also seen that it improves the range of motion (ROM) in joints. The elastic nature of this tape provides better traction and mobility in joints [14]. Applying kinesiology taping could improve kinesthetic and joint position sense by increasing the output of cutaneous mechanoreceptors [15]. Few articles emphasize that kinesiology taping application regains the function of the upper limbs, especially in increasing the functional range of motion (ROM), improving selective movements of fingers such as grasping and holding the glass or spoon, and providing stability to the shoulder. It has improved patients' physical fitness, gross motor function, and day-to-day activities [16]. The advantage of using kinesiology taping is that it reduces the pain of the muscle by raising the subcutaneous layer of the skin and reducing the chemoreceptor irritation [17]. Kinesiology taping also reduces the spasticity of muscles and can be considered an adequate therapeutic intervention for managing the control and coordination of muscles of the upper limb. The main drawback of using kinesiology taping is that there is not much evidence regarding the duration of tape application or the extent of stretching for its effects on patients with CP [18].

Robotic-assisted physiotherapy in treating patients with cerebral palsy

New methods have recently emerged for the rehabilitation and treatment of the motor disorders brought on by CP. One uses virtual reality and has shown improvements in the upper limb fine motor function and lower limb gross motor function in kids with CP. Lokomat, Innowalk, Robogait, and Waltbox-K are the robotic-assisted devices used to achieve the desired outcome in treating these children along with conventional physical therapies. Lokomat is one of the most commonly used robotic patient treatment devices [19]. This device has also proven effective in improving cerebellar-motor connectivity by stimulating sensory systems and proprioceptive receptors. Two types of gait devices useful in these children are end effectors and exoskeletons. End effectors are those devices that produce trajectory through the footplate by guiding the feet and swinging phase of gait. Exoskeletons are wearable devices for people walking on the ground and running on a treadmill [20]. Each pediatric Lokomat training requires a team of doctors, including neurologists, physiotherapists, and neuropsychomotricists. The main goal of robotic systems in treating these children is to achieve correct motor coordination of muscles. These devices can provide high-intensity and task-specific training, strengthening the muscles [21]. Hybrid robot-assisted limb gait training has a positive effect in improving the walking and posture of the children. Robotic-assisted training is also gaining popularity because of its qualitative aspects: the therapist can move freely without stopping the patient, and children get motivated and earn interest in participating in this intervention as they feel that the robot is a cool device. It increases the ROM in muscles and improves the fine movements of fingers to master the obstacles posed by daily activities. A hybrid robot-assisted limb is a rigid exoskeleton device that provides little action [22]. Robomorphism is the designed robotic equipment for developing an active lower limb. It is made up of elastic material, which makes it easy to wear and thus provides transparency. XPED2 is a passive lower limb exoskeleton for locomotion assistance; it is a lightweight structure used as a multiarticular extension to transmit energy between joints [23]. The primary advantages of utilizing these devices are the potential reduction of spasticity and enhancement of joint amplitudes, autonomy, muscle tone, and strength.

Given the novelty of these techniques, it is unclear how feasible and effective they will be in treating this condition and in this population [19].

Botulinum toxin intervention in the management of children with cerebral palsy 

In children with CP, botulinum toxin type A (BoNT-A) has become the most often used medical treatment over the past 20-25 years. It has been the most widely accepted intervention for treating CP patients. It significantly impacts large muscles of limbs, reduces spasticity, and increases range of motion with long-term use of at least 5-6 months. BoNT-A injections are usually safe for patients, but there are certain risk factors for ambulatory patients and more adverse systemic events in non-ambulatory patients.

Botulinum toxin is one of the most potent toxins used worldwide for treating CP patients. It is a standard intervention used in many countries [24]. Focal injection of this vaccine is effectively used to reduce spasticity of muscles and provide a better quality of life to patients of CP. Constant spasticity of muscles leads to contractures and deformity, thus hampering muscle growth, and their lengthening results in a short range of motion of muscles. The administration of this toxin enhances motor ability and improves posture. There are seven major serotypes (A-G) of clostridium botulinum, out of which botulinum toxin type A has a typical clinical use. Exocytosis is the process by which acetylcholine is released into synaptic clefts at the neuromuscular junctions, which ultimately causes changes in the electric potential of the membrane of muscles and excites the muscles [25]. Studies revealed that injection in gastrocnemius muscles recovers partially in 8-12 months. Before the administration of the injection, there is a shortening of muscle and equinus deformity at the ankle, but soon after the administration of the injection, acute atrophy of muscle is observed (decrease in spasticity and pain), and then, recovery of muscle occurs in 8-12 months [24].

Few articles suggested that both physical therapy and intense training strategies benefit children who use botulinum toxin injection. Using botulinum toxin, goniometric analysis showed a significant reduction in popliteal angle, resulting in an increased range of knee joint movements [26]. The dosage of this toxin is not standardized; rather, it is standardized as per specific muscle group. The dose depends on which muscle it is given, the muscle volume, and the degree of spasticity in muscles. The injection is given by identifying specific muscles to be delivered using ultrasonography with or without electromyography (EMG) guidance. Some injections available in the market are rimabotulinum toxin B, onabotulinum toxin A, abobotulinum toxin A, and incobotulinum toxin A. It is the most appropriate technique for locating muscles, as the effects of toxins are dependent on the uptake of the presynaptic membrane of the end plate of muscles [27]. Table 2 shows the different types of injections and their dosages used in treating CP.

**Table 2:** Generic name, brand name, and dosages of botulinum toxin injections used in treating CP CP: cerebral palsy

Generic name	Brand name	Units/vial
Rimabotulinum toxin B	Myobloc or Neurobloc©	2500, 5000
Onabotulinum toxin A	Botox®	100
Abobotulinum toxin A	Dysport®	500
Incobotulinum toxin A	Xeomin®	100

Physicians prefer EMG portable devices because handling and disinfecting smaller devices are easier; these devices are disinfected by UV-C light [28]. Botulinum toxin is not an isolated treatment of CP, and it is given along with other strategies such as orthotic intervention, physiotherapy, and casts [27]. Some articles suggested that after administering botulinum toxin injections, few systemic adverse events in patients with CP were observed, such as bowel and bladder incontinence and respiratory complications. Incontinence occurred within 2-5 days and was also resolved spontaneously without any medications within six weeks. However, respiratory complications can be due to anesthetic procedures or botulinum toxin injections, and it is more common in patients suffering from pseudobulbar palsies. Aspiration of food particles may lead to the death of patients due to uncoordinated movements of the sphincters of the larynx. Infusion of higher doses leads to emergency hospital admission for respiratory complications [29]. Respiratory tract infections, generalized pain in the body, laryngitis, bronchitis, weakness of muscles, fever, and seizures were also associated with adverse effects of this toxin [30]. To assess these adverse effects, physicians must evaluate the child and note the events occurring in patients via machines, signs, and symptoms. Recovery and the positive effects of drugs should be evaluated by professionals [31]. Effective benefits are achieved with minimal adverse effects with BoNT-A treatment. The main disadvantage of BoNT-A is that, despite its high efficacy in lowering muscular tone under dynamic settings, it has no impact on increasing the length range of force exertion or decreasing passive stiffness in the muscles [25].

Orthotic approach in treating patients with cerebral palsy

Crouch gait is one of the most common gait patterns in patients with CP. This gait requires more energy in muscles to walk. Walking deteriorates very quickly, leading to functional disability and deformity of the joints; hence, it should be managed adequately by using orthoses to prevent further deterioration. For managing this disability, ankle foot orthoses (AFOs) are preferred; they reduce the risk of further worsening by supporting the tibia, stabilizing the joint, and delivering better extension of the knee joint. Two types of AFOs recommended for these patients are hinged AFOs (HAFOs) and carbon spring AFOs (CAFOs). Orthoses restore the power of muscles and require less energy to walk, and they also provide adequate support to the limbs to walk properly on the ground [32]. The beneficial outcomes of orthoses are observed more in the sagittal plane than in coronal and horizontal planes [33]. As the knee joint is the main joint involved in the movement of limbs, orthoses are the most effective assisted devices used to increase the range of movements and support patients. Physicians' main goal is to make them walk in a natural and more comfortable environment without facing many difficulties.

There are certain disadvantages to using these devices: they are heavier, they need batteries to function and operate them, and patients also get uncomfortable using them [34]. The advantages of orthoses outweigh its disadvantages as they improve quality of life. Orthotic management plays a significant role in treating patients with CP to provide them with efficient walking. Orthoses provide proper alignment to the joints and form an overhead rehabilitation intervention for patients with CP [35]. Orthopedic surgeries are also required to manage crouch gait; these include lengthening the hamstrings by Z-lengthening methods and fractional lengthening of this muscle [36]. These surgeries and other interventions described earlier make a rehabilitation program for children with CP.

Recently, adjustable dynamic response AFOs (ADR-AFOs) have been introduced. ADR-AFOs have the advantage of providing better AFO adjustability, allowing variable ROM, and selectively supporting muscles that help in walking, such as the tibial anterior and gastrocnemius-soleus muscles. The primary drawback of using an assistive device is discomfort, as it might cause skin pressure, friction, or abrasions [34].

Table 3 shows a summary table of included studies.

**Table 3:** Summary table of included studies CP: cerebral palsy, NMES: neuromuscular electrical stimulation, ROM: range of motion, EMG: electromyography, AFOs: ankle foot orthoses

Author	Year	Findings
Jackman et al. [1]	2021	Best practices call for setting client-selected objectives and focusing intervention on the whole-task course of the goals rather than treating underlying impairments when attempting to enhance functional goals for children and young adults with CP.
Paul et al. [2]	2022	Different strategies and prevention methods have been identified to decrease the occurrence of this disorder.
World Health Organization [3]	2012	Interventions for these children primarily focus on training exercises, crucial real-world responsibilities, and specifically aiming for their full engagement within the community.
Vitrikas et al. [4]	2020	CP can also result in pain in the hip region or dislocation of the hip joint, balancing difficulties in the patients, and various dysfunctions.
O'Shea [5]	2008	The diagnosis of CP is typically based on the observations of the doctors and the history of achievement of milestones of children given by parents, such as neck holding/neck control, trunk control (rollover), sitting with support, sitting without support, crawling, walking, reflexes of deep tendons, and tone of muscles.
Novak et al. [6]	2020	Interventions include various physiotherapies, constraint-induced movement therapy, hippotherapy, botulinum toxin therapy, casting, correcting scoliosis using braces, umbilical cord therapy, and stem cell therapy.
Damiano et al. [7]	2009	The desired improvement will require a longer period to impact the muscles significantly. This requires immense training and support from the doctors, family, and trainers.
Fosdahl et al. [8]	2019	To maintain strength, stability, and dynamic control in both the mid-stance and terminal swing face, adequate active terminal knee extension is crucial.
Damiano et al. [9]	2009	Interventions such as ortho strategies, botulinum toxin injections, or other spasticity-reducing medications or surgeries with manual stretching techniques are effective. A team of doctors, such as physiatrists, neurologists, developmental pediatricians, and orthopedists, typically manage motor impairments in children with CP.
Moreau et al. [10]	2016	To increase gait speed in ambulatory children with CP, gait training is the most successful strategy.
Wang et al. [11]	2013	NMES is a common type of stimulation used along with other conventional treatments to improve motor function activities in children.
Karabay et al. [12]	2016	NMES also effectively controlled the posture in children of CP. It supports the spine and back muscles and helps in sitting. It also has some other advantages: it is a noninvasive technique, it improves muscles, and it also helps in proprioception.
Unger et al. [13]	2018	The kinesiology taping technique enhances the functions of the muscles and myofascial fibers of the skeletal muscles.
Uzunkulaoğlu et al. [14]	2018	Kinesiology taping offers remarkable improvements in managing pain, and it is also seen to improve the range of motion in joints. The elastic nature of this tape provides better traction and mobility in joints.
Iosa [15]	2014	The application of kinesiology taping could improve kinesthetic and joint position sense by increasing the output of cutaneous mechanoreceptors.
Kaya Kara et al. [16]	2014	Kinesiology taping application regains the function of upper limbs, especially in increasing the functional ROM, improving selective movements of fingers such as grasping and holding the glass or spoon, and providing stability to the shoulder.
Bartík et al. [17]	2022	Kinesiology taping reduces the pain of the muscle by raising the subcutaneous layer of the skin and reducing the chemoreceptor irritation.
Ortiz Ramírez et al. [18]	2017	Kinesiology taping reduces the spasticity of muscles and can be considered an adequate therapeutic intervention for managing the control and coordination of powers of the upper limb.
Llamas-Ramos et al. [19]	2022	Robotic-assisted devices are used to achieve the desired outcome for treating these children, along with conventional physical therapies. Lokomat is one of the most commonly used robotic devices for treating patients.
Bonanno et al. [20]	2023	The two types of gait devices useful in these children are end effectors and exoskeletons. End effectors are those devices that produce trajectory through the footplate by guiding the feet and swinging phase of gait.
De Luca et al. [21]	2022	The main goal of robotic systems in treating these children is to achieve correct motor coordination of muscles. These devices can provide high-intensity and task-specific training, strengthening the muscles.
Moll et al. [22]	2022	Robotic devices increase the range of movements in muscles and improve the fine movements of fingers to master the obstacles posed by daily activities. A hybrid assistive limb is a rigid exoskeleton device that provides little action.
Vertechy et al. [23]	2014	Robomorphism is the designed robotic equipment for developing an active lower limb. It is made up of elastic material, which makes it easy to wear and thus provides transparency.
Multani et al. [24]	2019	Botulinum toxin significantly impacts large muscles of limbs, reduces spasticity, and increases the range of motion with long-term use of at least 5-6 months.
Kaya Keles et al. [25]	2022	Exocytosis is the process by which acetylcholine is released into synaptic clefts at the neuromuscular junctions, which ultimately causes changes in the electric potential of the membrane of muscles and excites the muscles.
Fonseca et al. [26]	2018	Using botulinum toxin, goniometric analysis showed a significant reduction in popliteal angle, resulting in an increased range of knee joint movements.
Molenaers et al. [27]	2013	The injection is given by identifying specific muscles to be provided using ultrasonography with or without EMG guidance. It is the most appropriate technique for locating muscles, as the effects of toxins depend on the uptake of the presynaptic membrane of the end plate of muscles.
Yılmaz Yalçınkaya et al. [28]	2021	Doctors prefer EMG portable devices because handling and disinfecting smaller devices are easier. These devices are disinfected by UV-C light.
Naidu et al. [29]	2010	Aspiration of food particles leads to the death of patients due to uncoordinated movements of the sphincters of the larynx. Infusion of higher doses leads to emergency hospital admission for respiratory complications.
Papavasiliou et al. [30]	2013	Respiratory tract infections, generalized pain in the body, laryngitis, bronchitis, weakness of muscles, fever, and seizures were also associated with adverse effects of botulinum toxin.
Swinney et al. [31]	2018	Recovery of patients should be evaluated by doctors.
Borghi et al. [32]	2021	AFOs are preferred; they reduce the risk of further worsening by supporting the tibia, stabilizing the joint, and providing better extension of the knee joint.
Zhang et al. [33]	2017	The beneficial outcomes of orthoses are observed more in the sagittal plane than in coronal and horizontal planes.
Bayón et al. [34]	2023	Physicians' main goal is to make the children walk in a natural and more comfortable environment without facing many difficulties.
Melanda et al. [35]	2020	Orthotic management plays a significant role in treating patients with CP to provide them with efficient walking. Orthoses provide proper alignment to the joints and form overhead rehabilitation interventions for patients with CP.
Galey et al. [36]	2017	Orthopedic surgeries are also required to manage crouch gait; these include lengthening hamstrings by Z-lengthening methods and fractional lengthening of this muscle.

Study limitations and future research potentials

The main limitation found in this review is low methodology quality. Some studies that were selected had no control and randomization groups. Another limitation is that parents frequently talk about the difficulties children face in daily activities. Unfortunately, there are very few articles on the prevention of cerebral palsy and no appropriate data about using these interventions and their effectiveness. Children usually drop out of randomized controlled studies. The significant limitation of current AFOs is the adaptability of these devices to patients and the comfort of using these devices. The usage of BoNT-A injections was not properly recorded as the treatment duration is longer. Some of the symptoms told by children are not properly described; rather, it is subjective. There is no clear data available for using kinesiology taping, such as the duration of tape application or the extent of stretching.

Future studies should aim to determine the ideal training volume, frequency, intensity, and duration for these interventions and treatments that improve the quality of life. Clinical practice might be informed by using these recommendations to prescribe tailored treatments. The timing of such training should also be further investigated to maximize the effort, time, and financial resources given to patients with cerebral palsy. New AI technologies, such as ChatGPT, could help in individualizing and selecting treatment plans for CP patients. It is going to enhance the work, enhance prognosis, and help with management choice.

## Conclusions

The main goal in treating CP patients is to improve their lifestyle and make them independent to overcome life difficulties. Patients should adopt strategies and new interventions to improve their muscle activity, increase their range of motion, and provide confidence, and a comfortable environment should be provided for these children. The rehabilitation interventions used in treating CP are physiotherapies, orthotic approaches, corrective surgeries, casts, and robotic-assisted devices to improve their gaits. Different interventions have their own advantages and disadvantages and should be adopted accordingly. The benefits of these interventions require the assistance of many physicians, and these interventions also require energy and patience. Children with CP should adopt all these interventions to enhance their quality of life.
